# Severe course of Lyme neuroborreliosis in an HIV-1 positive patient; case report and review of the literature

**DOI:** 10.1186/1471-2377-10-117

**Published:** 2010-11-30

**Authors:** Nathalie D van Burgel, Mayke Oosterloo, Frank P Kroon, Alje P van Dam

**Affiliations:** 1 Department of Medical Microbiology, Centre of Infectious Diseases, Leiden University Medical Centre, Leiden, the Netherlands; 2 Department of Neurology, Leiden University Medical Centre, Leiden, the Netherlands; 3 Department of Infectious Diseases, Centre of Infectious Diseases, Leiden University Medical Centre, Leiden, the Netherlands; 4 Onze Lieve Vrouwengasthuis, Department of Clinical Microbiology, OLVG Hospital, Amsterdam, the Netherlands

## Abstract

**Background:**

Lyme Neuroborreliosis (LNB) in a human immunodeficiency virus (HIV) positive patient is a rare co-infection and has only been reported four times in literature. No case of an HIV patient with a meningoencephalitis due to LNB in combination with HIV has been described to date.

**Case presentation:**

A 51 year old woman previously diagnosed with HIV presented with an atypical and severe LNB. Diagnosis was made evident by several microbiological techniques. Biochemical and microbiological recovery during treatment was rapid, however after treatment the patient suffered from severe and persistent sequelae.

**Conclusions:**

A clinician should consider LNB when being confronted with an HIV patient with focal encephalitis, without any history of Lyme disease or tick bites, in an endemic area. Rapid diagnosis and treatment is necessary in order to minimize severe sequelae.

## Background

Lyme Neuroborreliosis (LNB) in a human immunodeficiency virus (HIV) positive patient is a rare co-infection and has only been reported four times [1-4]. All published cases are early presentations of Lyme disease and no report of a meningoencephalitis due to *B. burgdorferi *in an HIV patient has been made to date. We present a case of an HIV positive patient that presented with a severe LNB, without any previous sign of Lyme disease.

## Case presentation

A 51-year-old woman, diagnosed with HIV 10 years before, presented early spring 2006 at the outpatient clinic in the west of the Netherlands. She had noticed an altered gait that was progressive since three months. Strength and sensibility were unaltered, but there was paresthesia in both legs. She also had problems unbuttoning clothing with both hands. There was no complaint of headache, photophobia or visual changes. The medical history showed an anxiety disorder, hypertension and glaucoma. At presentation, the patient had been using HAART for six years (zidovudine, lamivudine and nevirapine) in combination with antihypertensive medication and a selective serotonin reuptake inhibitor. There was no indication for antibiotic prophylaxis. She had no history of tick bites, rash, erythema migrans or other signs of early or late-stage Lyme borreliosis. However, she frequently worked in her garden in an area where *B. burgdorferi *is endemic. Neurological examination revealed a bipyramidal walking pattern, an intention tremor of the posture and the hands, bilateral hyperreflexia in her legs and arms, a positive Hoffman-Trömner and a bilateral Babinski. There were no meningeal signs and all cranial nerve function was intact. RR was 190/113 and temperature was normal. Routine laboratory tests showed no signs of infection; blood leucocytes level was 5,9*10^9^/l. Plasma HIV RNA load was undetectable, CD4+ T lymphocyte count was 501/μl. A lumbar puncture was performed; the opening pressure was 28 cm H_2_O. The cerebrospinal fluid (CSF) showed a leucocytosis of 201/μl, 70% T-lymphocytes, 6% NK-cells, and 6% B-lymphocytes, glucose 2 mmol/l (serum glucose 6,2 mmol/l), protein 1,26 g/L. By isoelectric focussing oligoclonal IgG was detected intrathecally, but there was no evidence of a monoclonal B-cell population. Cytology and immunophenotyping of the intrathecal leucocytes were negative for haematological malignancies.

**Figure 1:**
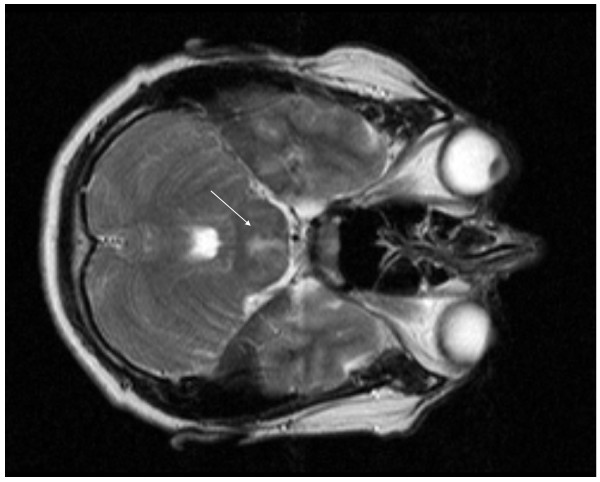
**DUAL TSE at presentation with a diffuse lesion located centrally in the pons**.

**Figure 2:**
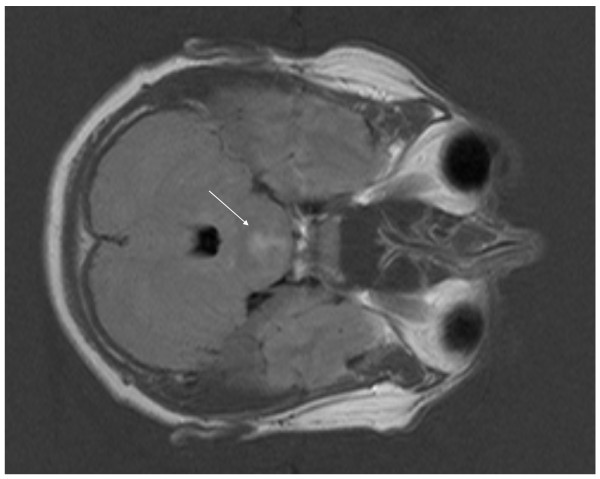
**FLAIR at presentation with a diffuse lesion located centrally in the pons**.

An MRI of the spine as well as brain was performed. No abnormalities were detected in the spine, but in the midline of the pontine region a hyperintense signal was seen on DUAL and FLAIR view (Figure 1-2). This lesion did not enhance under gadolinium.

CSF was examined and found PCR negative for HIV RNA, neurotropic viruses (Cytomegalovirus, Epstein Barr, Varicella zoster virus, Herpes simplex virus, JC virus), tuberculosis, toxoplasmosis, Bartonella and *Treponema pallidum*. CSF and serum serology was negative for *T. pallidum *and cryptococcal antigens. Serology for Bartonella, Babesia, Anaplasma and Ehrlichia showed no indication of an active infection. Culture was negative for cryptococcosis, tuberculosis and other common bacteria. Results for Lyme disease showed specific intrathecal IgG antibodies against *B. burgdorferi *in ELISA, no additional bands on blot were seen in CSF compared to serum. The intrathecal antibody index (AI) was positive (AI 19; cut off 0.3), indicating a specific production of antibodies to *Borrelia *in the CSF (IDEIA, Oxiod, UK). Also real-time PCR for *Borrelia burgdorferi *OspA conducted on the CSF was positive [5]. Serum antibodies against *Borrelia *were detected with the QuickELISA Borrelia C6 assay (Lyme index >10) (Immunetics, Boston, USA), and their presence was confirmed by a positive band for p100/83, VlsE, p41(i), p39 and DbpA on the RecomBlot Borrelia IgG assay (Mikrogen, Martinsreid, Germany). A serum from three years preceding this clinical presentation tested completely negative for antibodies against *B. burgdorferi*.

**Figure 3:**
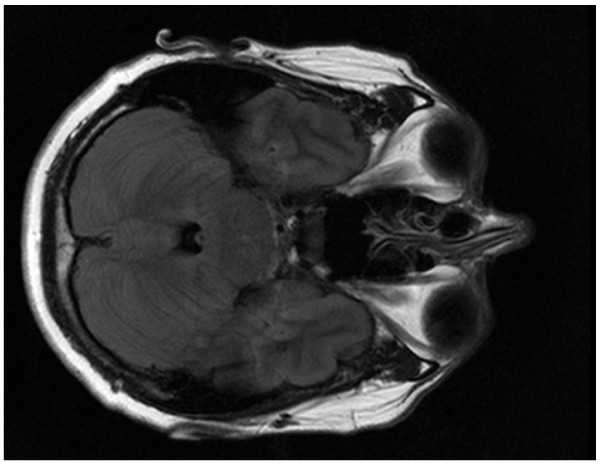
**FLAIR seven months after treatment, the lesion completely resolved**.

The diagnosis of a Lyme meningoencephalitis was made. The patient was treated intravenously with ceftriaxone 2 g/day for 1 month according to the EUCALB guideline. During the first week of treatment her clinical condition worsened. She was no longer able to walk independently and was forced to use a wheelchair. The cerebral MRI however showed decline of the hyperintense region at the end of intravenous treatment. An MRI performed one month later showed no abnormalities at all (Figure 3). In addition, posttreatment CSF showed a reduction in pleocytosis (7/μl), an absence of intrathecal specific antibodies against Borrelia and the real-time PCR for *OspA *on the CSF was negative. After a year of regular check up the patients' physical examination showed paraparesis of the right m. iliopsoas grade 4, right hamstrings grade 3, right footlifters degree 4 and hyperreflexia of both legs with Babinski signs. The bipyramidal walking pattern was still present. The patient was able to stand alone and walk with a cane. No further clinical improvement was detected at regular check up for four years.

## Discussion

We describe a case of a patient with an HIV infection and severe neurology and MRI lesions due to a *B. burgdorferi *central nervous system (CNS) infection. After treatment with ceftriaxone the laboratory parameters of infection disappeared along with the abnormalities on MRI, however the patient persistently suffered from severe verifiable sequelae.

Diagnosing Lyme disease using serology in patients with HIV is complicated due to reduced positive predictive value of serology in HIV patients [6]. Also, false positive serologic findings are described in patients with neurological infections with other spirochetes such as *Treponema pallidum *[7]. Present patients' clinical presentation was compatible with a localised cerebral infection with *B. burgdorferi*, confirmed by the positive serology, intrathecal specific anti-*Borrelia *antibody production and a positive real-time PCR for *B. burgdorferi OspA *on the CSF. The sensitivity of the *OspA *real-time PCR on CSF is only about 50% but the specificity is very high >99% [5]. In HIV positive patients where diagnosis of LNB by an indirect method such as serology is already compromised, a method where the microorganism is detected directly is of great value.

Little is known about the course of LNB in HIV patients. Animal models have shown that CD4 + T-cells facilitate clearance of *B. burgdorferi *[8]. In recent studies the importance of CXCL13 in B-cell recruitment in patients with LNB has been suggested [9]. In HIV infection the levels of CXCL13 in serum are elevated and the receptor CXCR5 on B-cells is down regulated causing impaired trafficking of B-cells [10]. Inadequate humoral response could lead to accelerated progression of LNB. In animal experiments immunodeficiency leads to higher spirochete burdens and higher infectivity of *B. burgdorferi *[11]. In contrast, reports from a study where Lyme borreliosis patients were treated with immunosuppressive agents, no significant effect on clinical course and response to treatment was observed [12]. *T. pallidum *is a spirochete and more is known about disease course in syphilis HIV co-infection. Disease course is altered in *T. pallidum *and HIV co-infections; there is a higher rate of asymptomatic infection, a faster progression to secondary disease which is often more aggressive with a significant predisposition for the development of neurological complaints. However, after treatment HIV positive patients recover as well as the HIV negative population [13,14]. In co-infections of HIV and *Leptospira *species. a more fulminant disease course has also been suggested [15]. For LB and HIV co-infections no such synergistic complications have been described in patients to date.

Despite the high incidence of HIV, co-infection with *B. burgdorferi *is not reported very often [1-4]. The low incidence of HIV LNB co-infections can not be easily explained. In HIV infection antibiotic prophylaxis is sometimes prescribed but none of the reported cases of HIV patients with LNB used antibiotic prophylaxis. Prophylaxis usually consists of cotrimoxazole, which is ineffective against *B. burgdorferi *which renders a positive effect of antibiotic prophylaxis on the development of Lyme disease unlikely [16]. There is no data about anti-borrelia activity for anti-retroviral mediaction but an association seems unlikely because three of the five described patients were using anti-retroviral medication. All reported LNB HIV patients' results have been summarized in table 1 and table 2. Case 1 had had an EM and presented with a rather classical course of a bilateral facial palsy shortly after noticing the EM, rapidly improving on IV ceftriaxone treatment. HIV serology was found positive during workup for the cause of his bilateral facial palsy, CD4+ count was decreased at that time. Case 2 had a low CD4+ count with progression to neuroborreliosis, despite treatment for his recent early LB. Serology in CSF and serum was clearly positive for an early LNB. After IV treatment with ceftriaxone he recovered completely. Case 3 had a very low CD4+ count and was the only case that met the criteria for AIDS. He primarily showed a slow seroconversion although this was only determined by indirect immunofluorescence and not by ELISA or Western Blot. Four months after presentation with a painful radiculitis he had low detectable Ig titers against *Borrelia *by ELISA and Western Blot. He recovered completely. Case 4 had a moderately low CD4+ count and had a classical course of EM, followed by malaise and a facial palsy with signs of early LNB in serology of CSF and serum. He responded well to treatment with IV cefotaxime. Case 5 the present case had a moderately low CD4+ T-lymphocyte count and had rapid progression to a meningoencephalitis. This is the only case described to date of an HIV patient with a Lyme meningoencephalitis. The course of disease was rapid and atypical with a primary presentation of altered gait due to Lyme meningoencephalitis, which is an uncommon presentation of neuroborreliosis, found in only 3-5% of patients with LNB [17]. Diagnosis was made obvious due to positive PCR combined with positive IgG serology in CSF and serum. After treatment microbiological response was rapid. AI returned negative after two months, which is a rapid decline but not uncommon in literature [18]. Despite this rapid improvement biochemically and microbiologically severe sequelae remained. Posttreatment sequelae are rare. Patients with posttreatment sequelae have complaints of fatigue, cognitive impairment and paresthesia but sequelae are rarely as severe after a relatively short duration of illness as in this case [17,19,20]. Although this patient had complaints for only three months, it is likely that earlier recognition and treatment of LNB in this patient would have led to less neurological damage and therefore to better recovery.

**Table 1:** Clinical data from all patients with LNB and HIV reported in literature.

Case no.*	Anti-retroviral	Antibiotic prophylaxis	Skin manifestations	Presentation	Treatment	Clinical recovery
1	none	none	Annular erythematous lesion	Several weeks later: fever, bilateral facial palsy	IV ceftriaxone 2 g/day 14 days	After treatment vast improvement 2 months, complete recovery
2	Zidovudine	none reported	Erythema	Headache, painful limbs, weight loss, pneumonia	Primairily: PO Azithromycin	Progression to neuroborreliosis
	Saquinavir				1 day 500 mg, 4 days 250 mg	
	Zalcitabine			2 weeks later: fever, diplopia	IV ceftriaxone 2 g/day 14 days	Improved rapidly
3	Zidovudine	none reported	Erythematous lesion	4 weeks later:: radiculitis	IV ceftriaxone 2 g/day 15 days	Complete recovery
	Didanosine					18 months, no relapse
4	none	none reported	Multiple	Fever, chills, arthralgias	IV ceftotaxime 2 g TID 21 days	After treatment mild facial palsy
			Maculous erythemas	2 weeks later left facial palsy		6 months, slight hypokinesia face
5	Zidovudine	none	None	Altered gait for months	IV ceftriaxone 2 g/day 1 month	After treatment severe sequelae
	Lamivudine					3 years, no relapse
	Nevirapine					

*Case 1[1], 2[2], 3[3], 4[4], 5 this report.

**Table 2:** Laboratory data from all patients with LNB and HIV reported in literature.

Case no.*	Age	Sex	Yrs HIV	Blood CD4+ count (/μl)*	CSF cell count (/μl)	Protein (g/l)	CT/MRI	Serum Serology	CSF Serology
1	39	M	0	386	30	1,02	nd	IgG +	IgG +
								ELISA/WB	ELISA/WB
2	39	M	1	250	496	3,62	nd	IgM +/IgG -	IgG +
3	50	M	10	70	'aseptic meningitis'		nd	ELISA IgG + (month 4)	ELISA IgG + (month 4)
								WB IgG +	WB IgG +
								IF Negative >4 months	
4	46	M	16	426	416	3,02	normal	IgM +/IgG +	IgM +/IgG +
								ELISA/WB	ELISA/WB
5	51	F	11	501	200	1,26	abnormal	IgM -/IgG +	IgM -/IgG +
								ELISA/WB	ELISA/WB

*Case 1[1], 2[2], 3[3], 4[4], 5 this report. Serology was performed by ELISA, Western blot (WB), or indirect immunofluorescence (IF). (nd = not done)* normal range peripheral blood; case 1: 580-1570/μl, case 3: 600-1000/μl, case 5: 560-1490/μl

## Conclusions

A clinician should consider LNB as a rare possible cause of focal encephalomyelitis in an HIV patient, without any history of Lyme disease or tick bites in an area endemic for Lyme disease. Diagnosis of LNB can be compromised in HIV co-infected patients, however when applying optimal serological and molecular diagnostic techniques confirming LNB is imminent. This report raises the possibility that LNB might take a more severe course in immunocompromised patients, such as those with HIV infection.

## Consent

Written informed consent was obtained from the patient for publication of this case report and any accompanying images. A copy of the written consent is available for review by the Editor-in-Chief of this journal.

## Competing interests

Financial disclosure related to research covered in this article: none for all authors

## Authors' contributions

Clinical work-up and literature search were performed by NDvB, MO, FPK, APvD. All authors made critical contributions to the paper and approved the final manuscript.

## Pre-publication history

The pre-publication history for this paper can be accessed here:

http://www.biomedcentral.com/1471-2377/10/117/prepub
